# Supplementary material for: Therapeutic interfering particles exploiting viral replication and assembly mechanisms show promising performance: a modelling study
Source: Sci Rep. 2021 Dec 13;11:23847. doi: 10.1038/s41598-021-03168-0 (PMC8668974; doi:10.1038/s41598-021-03168-0)
Supplement: Supplementary file 1 — Supplementary Information. [file 41598_2021_3168_MOESM1_ESM.pdf]

Supplementary Information

This file contains the supplementary material to support the manuscript: "*Therapeutic Interfering Particles Exploiting Viral Replication and Assembly Mechanisms Show Promising Performance: A Modelling Study*", submitted to the Scientific Reports journal.

Farzad Fatehi[1,2,+], Richard J Bingham[1,2,3,+], Pierre-Philippe Dechant[1,2,4,+], Peter G Stockley[5,*], and Reidun Twarock[1,2,3,*]

[1] *York Cross-disciplinary Centre for Systems Analysis, University of York, York YO10 5GE, UK*
[2] *Department of Mathematics, University of York, York YO10 5DD, UK*
[3] *Department of Biology, University of York, York YO10 5NG, UK*
[4] *School of Science, Technology Health, York St John University, York YO31 7EX, UK*
[5] *Astbury Centre for Structural Molecular Biology, University of Leeds, Leeds LS2 9JT, UK*
[*] *Corresponding Author: rt507@york.ac.uk*
[+] *these authors contributed equally to this work*

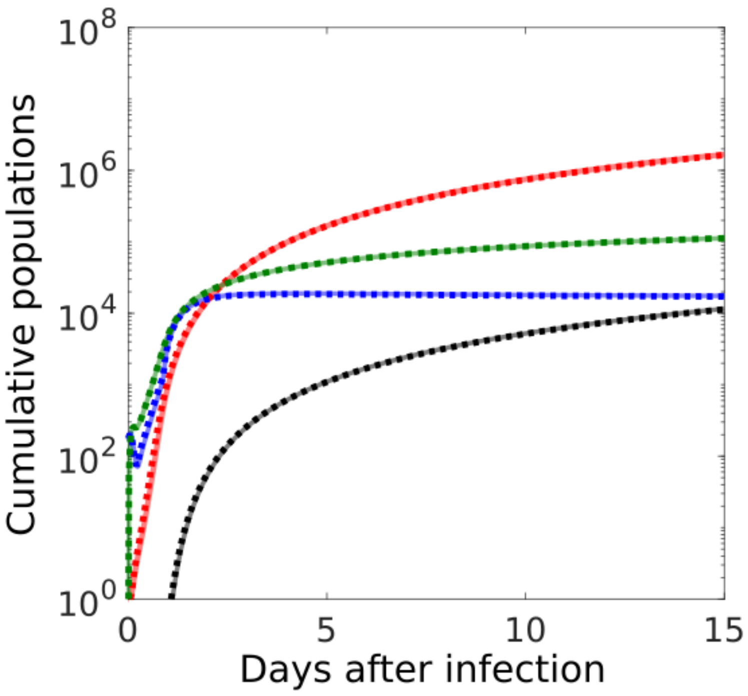

Figure S1: Comparing of the mean over 250 simulations of the Gillespie algorithm with the ODE system outcomes. Solid and dotted curves indicate the outcomes of the ODE system and Gillespie algorithm, respectively. The black, red, green and blue show the number of released virions, HCV RNAs, structural proteins and NS3 proteins. This figure shows that the mean over the Gillespie algorithm is same as the ODE model.
